# Redressing COVID-19 vaccine inequity amidst booster doses: charting a bold path for global health solidarity, together

**DOI:** 10.1186/s12992-022-00817-5

**Published:** 2022-02-22

**Authors:** Sudhan Rackimuthu, Kapil Narain, Arush Lal, Faisal A. Nawaz, Parvathy Mohanan, Mohammad Yasir Essar, Henry Charles Ashworth

**Affiliations:** 1 Father Muller Medical College, Mangalore, Karnataka India; 2 Nelson R. Mandela School of Medicine, University of KwaZulu-Natal, Durban, South Africa; 3 Department of Health Policy, London School of Economics and Political Science, London, UK; 4 Women in Global Health, Washington, DC, USA; 5 Community and Civil Society Representative, ACT Accelerator, London, UK; 6 College of Medicine, Mohammed Bin Rashid University of Medicine and Health Sciences, Dubai, United Arab Emirates; 7 Faculty of Medicine, Medical University Sofia, Sofia, Bulgaria; 8 Kabul University of Medical Sciences, Kabul, Afghanistan; 9 Harvard Medical School, Boston, MA USA

**Keywords:** COVID-19, COVID-19 vaccine booster, Global health, Health equity

## Abstract

**Background:**

With large swathes of the world’s population—majority clustered in low- and middle-income countries—still yet to receive the minimum of two doses of the COVID-19 vaccine; The need to address the failures of international solidarity to equitably distribute COVID-19 vaccines is now more urgent than ever to help curb the pandemic and prevent future variants. However, many high-income countries have adopted a “me first” approach, proceeding to offer COVID-19 booster doses to their entire populations, including those at least risk of severe illness, whilst the rest of the world is left unvaccinated or partially vaccinated with one dose for even their most vulnerable communities.

**Main body:**

COVID-19 vaccine inequity places the health of the global population at risk and exacerbates socio-economic repercussions, especially in low- and middle-income countries. Initiatives launched to combat vaccine inequity such as the Fair Allocation Framework for the COVID-19 Vaccines (COVAX) have been unsuccessful as several governments, primarily from high-income countries, have scaled down their contributions to the initiative. Furthermore, COVAX has not seriously engaged with the Access to COVID-19 Tools (ACT) Health Systems Connector, as was originally intended, leading to crucial health systems components critical to vaccine delivery to be overlooked. Several strategies can be employed to help achieve the desired global immunization goals, such as Intellectual Property waivers, increased donations, and activation of new COVID-19 vaccine manufacturing hubs. In addition, continued advocacy for vaccine equity by all involved and affected stakeholders, as well as critical amendments to existing or upcoming legislation and funding mechanisms will help address the shortcomings of current inequitable vaccine distribution.

**Conclusions:**

Global solidarity and collective action through pandemic governance mechanisms are urgently needed to ensure vaccine equity. These interventions are vital to rapidly mitigate ongoing health and humanitarian crises and ultimately curb the pandemic, sooner rather than later.

## Background

On the first day of the 74th World Health Assembly, Dr. Tedros Ghebreyesus, World Health Organization (WHO) Director-General stated, “No one is safe until everyone is safe.” His assertion underscored the urgency and necessity for international solidarity and collaboration to end the COVID-19 pandemic. Instead, many wealthy nations adopted a pernicious “me first” approach with respect to vaccine distribution, resulting in devastating consequences for the global population [1].

Public health officials have estimated roughly 75% of the world must be completely vaccinated to mitigate the COVID-19 pandemic [2]. However, as of January 13, 2022, a staggering 36 WHO Member States (countries) have vaccinated less than 10% of their populations and 88 below 40% even with Fair Allocation Framework for the COVID-19 Vaccines (COVAX) delivering it’s one billionth COVID-19 vaccine dose [3]. The case in Africa seems to be even more concerning with more than 85% of its total population having still not received even a single dose of the vaccine [4]. The vast majority of the double-vaccinated populations reside in high-income countries (HICs), while only 10% of individuals from low-income countries (LICs) have received at least one dose as of January 30, 2022 [5]. Merely 10 nations had administered more than three-quarters of all doses as of April 2021 [2], creating unconscionable challenges for remaining nations trying to procure lifesaving vaccines directly from manufacturers or through multilateral initiatives [6]. Unsurprisingly, vaccination supply has therefore been strikingly inadequate across low- and middle-income countries (LMICs).

Despite this backdrop of stark inequity^1^, wealthier countries have added insult to injury, offering COVID-19 booster doses not just to the elderly and immunocompromised, but across their entire general populations (often regarded as low-risk) [7]. HICs continue to rapidly mobilize resources to amplify booster campaigns, close borders, and pay high costs to procure additional doses of COVID-19 vaccines, thus resulting in vaccine nationalism. While these efforts appear fair and logical at the domestic level for these HICs, they do not exist in a vacuum. The pandemic and its variants do not respect borders. These efforts directly undermine solidarity for global health and put the pandemic response at greater risk of floundering. As long as HIC leaders allow COVID-19 vaccine inequity to endure by using up limited supply to deliver booster doses to their healthiest communities while the rest of the world is forced to wait for vaccines for their most vulnerable groups, the health and safety of all of us will remain at grave risk. Prolongation of the pandemic because the majority of the world’s population is unable to get vaccinated provides greater opportunity for viral transmission and mutation. This drives the emergence of new variants of concern, which may be more transmissible and virulent, like the devastating Delta and Omicron variants.

### Main Body

Early in the pandemic, several initiatives were launched to avoid the disaster of inequitable access to vaccines, including the Fair Allocation Framework for the COVID-19 Vaccines under COVAX, which forms one of the four pillars of the Access to COVID-19 Tools (ACT) Accelerator. Through COVAX, participating governments had pledged to ensure all countries reached the 20% vaccine coverage target before further scaling up efforts within their own borders. However, in flagrant disregard for this agreement, many HICs continued to hoard vaccine doses amidst an already-scarce supply, leaving COVAX under-resourced and LMICs with little to no access to vaccines. This ultimately widened the vaccine inequity gap and led to preventable deaths [8]. Though some HICs have stated their intention to donate residual supply to COVAX for distribution to qualified LMICs, progress to date has been grossly insufficient and deeply troubling.

Meanwhile, several HICs such as France, Germany, Israel, UK, USA, and UAE have utilized their vast vaccine inventories to begin administering additional booster doses to even low-risk groups while millions around the world including frontline health workers, the elderly, and the immunocompromised are yet to receive their first dose [9–11]. To compound this, reports of vaccine wastage in countries like the USA further rub salt in the wound, when many LMICs are trying every possible avenue to secure doses [12]. This unfolding crisis is only the latest in a long history of examples that show our current global health architecture with its existing regulations and guidelines are glaringly ineffective in safeguarding equity—vaccine equity being just one example. Urgent implementation of bold, novel, and especially inclusive approaches must become a pressing priority.

Notably, COVAX has not seriously engaged with the ACT Accelerator’s Health Systems Connector, as was originally intended, nor meaningfully acknowledged the in-country inequities in accessing COVID-19 vaccines. This signifies that vital health systems components to support vaccination have been routinely overlooked, including adequate numbers of trained and protected health workers to deliver vaccines, disaggregated data and information systems to identify bottlenecks and gaps in vulnerable communities, responsive communication based on intersecting social positionalities (including gender and sociocultural factors), and provisions to avoid disruptions to essential health services given the high demand for resources diverted to support vaccination campaigns.

Vaccine inequity will continue to have significant and enduring socio-economic repercussions in LMICs without urgent measures to boost supply and guarantee impartial access to medical countermeasures including COVID-19 vaccines and therapeutics. The ripple effects of vaccine inequity impact food security, education, travel, and commerce, especially in LMICs where infrastructure and resources may already be insufficient. This means that the longer it takes to achieve global vaccine equity, the longer socioeconomic and health crises will likely abound in LMICs. The diversion of a country’s limited resources towards mounting an adequate COVID-19 response curtails progress towards social and economic stability with far-reaching consequences. In fact, the United Nations Development Program predicted that the economic recovery rate will be faster in countries with higher vaccination rates, with an estimated USD 7.93 billion increase in global Gross Domestic Product (GDP) for every million people vaccinated [13].

In LMICs where vaccination rates are poor, the path to recovery will be lengthy, arduous, and rocky until equitable access to vaccines is guaranteed. To address these challenges, it is important to understand the factors perpetuating vaccine inequity. This global health and humanitarian crisis partly exists due to financial strengths of today’s HICs, enabling them to purchase a surplus of additional doses of vaccines. Moreover, astute bargaining between HICs and pharmaceutical industries further complicates the situation for LMICs. Long-standing relationships between vaccine manufacturers and HICs meant wealthier nations were well-equipped to negotiate and procure vaccines swiftly, further aided by the attractively higher prices that HICs were willing to pay. In addition, there is currently no regulated, standardized price for the procurement of COVID-19 vaccines, thus tragically enabling manufacturers to prioritize profits over lives. The anemic response and lack of precise, timeous, and definitive interventions from the World Trade Organization (WTO) with regards to a possible Trade-Related Aspects of Intellectual Property Rights (TRIPS) waiver was a missed opportunity at a time when few options were left on the table.

This unfolding catastrophe is far from coincidental. This is yet again another case, historically entrenched in the legacy of colonialism as well as a rigid capitalist and sociopolitical machinery where the rich nations become richer (and incidentally healthier) at the expense of poorer nations. Vaccine inequity seems to be preserved by an iniquitous arrangement whose system exists to benefit through discriminating the lives of one group of people over another: Global North above South, West over East, HICs over LMICs, and White communities above Black, Asian and Latino ones.

Hence, to reach the desired global immunization goals, multiple strategies must be employed to ensure HICs and pharmaceutical industries collaborate to expedite the manufacturing process of vaccines, and re-direct currently earmarked booster doses to vaccine-deprived LMICs. First, Intellectual Property (IP) waivers, including the TRIPS agreement waiver for COVID-19 vaccines, would assist LMICs to purchase and even produce their own vaccines on their own terms. Second, to emancipate production for global health, international organizations should develop and support vaccine manufacturing hubs in regions hit hard by the pandemic. International and multilateral finance institutes have a particularly important role to play in facilitating timely access to funding and resources, as well as promoting economic stimulation domestically. Finally, both local and international organizations need to continuously advocate and hold leaders accountable to ensure vaccine manufacturing is amplified and equitable distribution is achieved.

Notably, it is vital that the architects of major initiatives developed in the wake of COVID-19—particularly HIC donor governments and international organizations like the WHO and WTO—adapt, unlearn, and relearn from local communities especially in LMICs, in order to mitigate the drivers of vaccine inequity, both for short-term pandemic response and long-term health security. Subsequent reviews of the COVAX Strategy should ensure a strong focus on domestic health inequities and scaling up foundational health systems interventions to support vaccine delivery. Additionally, the G20-proposed Financial Intermediary Fund for Pandemic Preparedness and Response and Global Health Threats Board should include civil society and community voices as well as clear processes to rapidly finance and guide integrated and resilient responses to mitigate the pandemic, including future vaccination campaigns [14].

With negotiations for the pandemic treaty and International Health Regulations (IHR) revisions currently underway [15], WHO Member States must prioritize the inclusion of robust accountability mechanisms that go beyond traditional barriers in global health governance that fuels fragmentation and inequities. They should instead enable robust and transparent engagement of multisectoral stakeholders, including trade, private sector, finance institutions, and community advocacy groups. The multifaceted barriers to vaccine inequity were unapologetically devised by colonial powers who still continue to reap the benefits by systemically excluding the invaluable narratives and grassroots experiences of communities in LMICs, thereby discounting, and minimizing their genuine challenges. If we fail to use the current COVID-19 pandemic as a catalyst to make necessary reforms which includes bolstering health equity, the world’s populations—from HICs and LMICs alike—will be at far greater risk of adverse consequences stemming from either climate change, natural disasters, or the next great pandemic.

## Conclusions

Global solidarity is needed now more than ever. Heads of state of HICs and pharmaceutical industries alike must not only be deeply cognizant about the adverse consequences of a protracted pandemic but held accountable on a social, medical, legal, ethical, and moral level, as the ramifications of vaccine inequity are far-reaching and ultimately devastating for all populations. Providing equitable access to vaccinations through donations, IP waivers, and activation of manufacturing hubs are key interventions in reducing morbidity and mortality as well as improving the quality of life for communities around the world. Furthermore, collective action through solidarity-based pandemic governance mechanisms, rooted in the principles of postcolonialism and a right to health, are urgently needed to ensure vaccine equity. These interventions are vital to rapidly mitigate ongoing health and humanitarian crises and ultimately end the pandemic, sooner rather than later.

## Data Availability

Not applicable.

## Abbreviations

ACT: Access to COVID-19 Tools
COVAX: COVID-19 Vaccines Global Access
COVID-19: Corona Virus Disease-2019
GDP: Gross Domestic Product
HIC: High-Income country
IHR: International Health Regulations
IP: Intellectual Property
LIC: Low-Income country
LMIC: Low-and Middle-Income country
TRIPS: Trade-Related Aspects of Intellectual Property Rights
WHO: World Health Organization
WTO: World Trade Organization

## Footnotes

1 Inequities: Inequities are unfair systematic differences present in different population groups. Inequities commonly have significant social, health and/or economic repercussions both to individuals as well as societies.
